# Huaier suppresses cell viability, migration and invasion in human non-small cell lung cancer via lncRNA DLEU2/miR-212-5p/ELF3 axis

**DOI:** 10.7150/ijms.89308

**Published:** 2024-01-01

**Authors:** Tangwei Wu, Shuiyi Liu, Weiqun Chen, Dan Zhao, Zhongxin Lu

**Affiliations:** 1 College of Pharmacy, Hubei University of Chinese Medicine, Wuhan 430065, China.; 2 Department of Medical Laboratory, The Central Hospital of Wuhan, Tongji Medical College, Huazhong University of Science and Technology, Wuhan 430014, China.; 3 Key Laboratory for Molecular Diagnosis of Hubei Province, The Central Hospital of Wuhan, Tongji Medical College, Huazhong University of Science and Technology, Wuhan 430014, China.; 4 Cancer Research Institute of Wuhan, The Central Hospital of Wuhan, Tongji Medical College, Huazhong University of Science and Technology, Wuhan 430014, China.; 5 School of Laboratory Medicine, Hubei University of Chinese Medicine, Wuhan 430065, China.

**Keywords:** Huaier, NSCLC, ncRNA, migration, invasion

## Abstract

Accumulating studies suggest that Huaier exerts anti-tumor effects through intricate mechanisms. Despite extensive research on its efficacy in lung cancer, further investigation is required to elucidate the molecular mechanism of Huaier. The involvement of long noncoding RNAs (lncRNAs) in the anti-lung cancer effects of Huaier remains unknown. In this study, we found Huaier suppressed cell viability, migration and invasion in non-small cell lung cancer (NSCLC) cells. LncRNA sequencing analysis revealed Deleted in lymphocytic leukemia 2 (DLEU2) to be significantly downregulated in Huaier-treated NSCLC cells. Furthermore, DLEU2 silencing was observed to suppress NSCLC progression, while DLEU2 overexpression attenuated the anti-tumor effects of Huaier in NSCLC, thereby promoting cell viability, migration and invasion of NSCLC. The ceRNA role of DLEU2 had been demonstrated in NSCLC, which directly interacted with miR-212-5p to rescue the repression of E74 Like ETS Transcription Factor 3 (ELF3) by this microRNA. Additionally, Huaier was found to regulate the expression of miR-212-5p and ELF3. Functionally, miR-212-5p inhibitor or ELF3 overexpression reversed the effects of DLEU2 silencing or Huaier treatment, resulting in increased colony formation, migration and invasion in NSCLC. Taken together, these results illuminate the mechanism underlying Huaier's anti-tumor effects via the DLEU2/miR-212-5p/ELF3 signaling pathway, which offers novel insights into the anti-tumor effects of Huaier and constitutes a promising therapeutic target for the treatment in NSCLC.

## Introduction

As indicated by the 2023 cancer statistics, lung cancer is a prevalent malignancy with the highest mortality rate^1^. Lung cancer can be categorized into two main types: non-small cell lung cancer (NSCLC) and small cell lung cancer (SCLC). NSCLC accounts for approximately 85% of all lung cancer cases, encompassing subtypes such as lung adenocarcinoma, squamous cell carcinoma, and large cell lung cancer. Chemotherapy remains the primary treatment modality for NSCLC patients^2^. However, many effective chemotherapeutic agents are associated with systemic toxicity and multidrug resistance, which significantly limits their efficacy^3^. Several studies have demonstrated that combining conventional therapy with adjuvant Traditional Chinese Medicine (TCM) can mitigate treatment-related adverse effects, enhance the quality of life and reduce mortality in cancer patients^4^, ^5^.

Trametes robiniophila Murr is an official fungus that originates from the trunks of trees and has been utilized as TCM for approximately 1600 years^6^. The hot aqueous extraction of Trametes robiniophila Murr (Huaier) or granule is a commonly used pharmaceutical preparation. In recent years, an increasing number of studies have been conducted to explore the intricate mechanisms underlying the anti-tumor effects of Huaier^6^, ^7^. The available evidence indicated that Huaier could induce immunogenic cell death^8^, inhibit cell growth, migration and energy metabolism^9^, induce apoptosis^10^, activate cell autophagy induced ferroptosis^11^, enhance the sensitivity to chemotherapeutic agents^12^, and so on. Although some research have been performed on the anti-lung cancer mechanisms of Huaier^13^, ^14^, further research is imperative.

Long noncoding RNAs (lncRNAs) are a class of noncoding RNAs that exceed 200 nucleotides in length. LncRNAs have been reported to act as oncogene or tumor suppressor in diverse biological processes^15^-^17^. With the development of RNA sequencing, more and more lncRNAs have been found to be dysregulated in lung cancer^18^. Several lncRNAs, such as LINC01140^19^, HNF1A-AS1^20^ and SNHG6^21^ have been demonstrated to be upregulated in lung cancer. Other lncRNAs, such as ZNRD1-AS1^22^ and HITT^23^ are downregulated in lung cancer. These lncRNAs play a crucial role in the pathogenesis and therapeutic strategies of lung cancer. There are reports shown Huaier suppresses breast cancer progression via linc00339/miR-4656/CSNK2B signaling Pathway^24^. Since lncRNAs have been implicated in the anti-breast cancer effects of Huaier, it is worth investigating whether they also play a role in the anti-NSCLC effects of Huaier. So far, there have been no reports indicating the involvement of lncRNAs in Huaier's anti-NSCLC mechanisms.

In this study, we examined the expression patterns of lncRNAs in non-small cell lung cancer cells treated with Huaier and untreated controls using RNA sequencing, and subsequently investigated the potential role of specific lncRNA in mediating Huaier's anti-tumor effects. Our data provides innovative insights into the anti-tumor effects of Huaier and presents a promising therapeutic target for NSCLC treatment.

## Materials and methods

### Cells and cell culture

The human non-small cell lung cancer cell lines (A549, H1299), and the normal lung epithelial cell line BEAS-2B, and the human embryonic kidney (HEK) 293T cell were all obtained from American Type Culture Collection (ATCC). A549, H1299, BEAS-2B and HEK-293T cell lines were cultured in Dulbecco's modified Eagle's medium (DMEM, Gibco, Grand Island, NY, USA) supplemented with 10% fetal bovine serum (FBS, PAN, Eidenbach, Bagoria, Germany). Cells were grown in an incubator with 5% CO_2_ at 37 °C.

### Preparation of Huaier

Huaier was provided by Qidong Gaitianli Pharmaceutical Co., Ltd (Jiangsu, China). The stock solution of Huaier (100 mg/mL) was prepared for use as previously described 25.

### Cell counting kit-8 (CCK8) assay

Cell viability was detected by CCK8 assay. 8000 A549 cells/well and 5000 H1299 cells/well were respectively seeded into 96-well plate and cultured overnight. Then, medium was replaced with the fresh medium containing specific concentrations of Huaier (0, 1, 2, 4, 8, 12, 16 or 20 mg/mL). After incubation for 48h and 72h, the supernatant was removed and the 100 μL medium containing 10% CCK8 reagent (Beyotime, Nantong, China) was added to each well. Absorbance values were determined by an EnSpire multimode reader (PerkinElmer, Waltham, MA) at 450nm.

### Colony formation assay

For colony formation assay, A549 and H1299 cells (500 cells/well) were seeded in 12-well plates and cultured for ten days. The formed colonies were washed with phosphate-buffered saline (PBS) twice and stained with 0.1% crystal violet (Beyotime). A colony containing at least 50 cells was counted as one colony to represent the malignant viability of a single cell.

### Wound healing assay

For cell migration assay, A549 and H1299 cells were seeded into 6-well plates (3×10^5^ cells/well) and cultured for 24 h. A sterile 200 μl pipette tip was used to scratch the confluent cell monolayer vertically to make an artificial wound. Then, cells were washed with PBS twice and maintained in DMEM containing 2% FBS or various concentrations of Huaier. The wound closure images (magnification, 100×) were captured at 0 h and 48 h by IX81 microscope (Olympus, Tokyo, Japan). The wound healing rate was analyzed by Image J software.

### Transwell assay

For cell invasion assay, transwell chamber (Corning, NY, USA) with 8 µm pore filters was used. The upper chamber was precoated with 100 μl 10% Matrigel (BD Biosciences, CA, USA). After appropriate treatment, 4×10^4^ cells were resuspended in 100 μl serum-free medium and seeded into the upper chamber. DMEM containing 15% FBS was added into the lower chamber. After incubation for 24 h, the non-invaded cells on the surface of the upper chambers were removed by cotton swab. The cells that invaded to the basal side of the membrane were fixed with 4% formaldehyde (Beyotime) and stained with 0.1% crystal violet (Beyotime). Five fields of view at 100× magnification of the stained cells were selected randomly and calculated the mean using IX81 microscope (Olympus).

### lncRNA sequencing

A549 and H1299 were treated with 0 mg/mL and 4 mg/mL Huaier for 48 h. Then, the cells were collected and frozen in TRIzol (Invitrogen, Carlsbad, CA, USA). The experiment was repeated three times. Next, we pooled the cell lysate generated from three respective times of the same group for further RNA extraction. Total RNA was quantified and qualified using Agilent 2100 bioanalyzer (Thermo Fisher Scientific, MA, USA). Subsequently, the cDNA library was created and assessed before sequencing. Illumina (HiSeq X-Ten) sequencing was then performed to profile the expression of lncRNA according to the manufacturer's protocol. The raw data was analyzed using HISAT comparison software. Fold change was calculated to identify the deregulated lncRNAs. Heml software was used to draw the heatmap according to the lncRNA level in NSCLC cells.

### RNA isolation and qRT‑PCR

The total RNA was extracted from NSCLC cells with TRIzol reagent (Invitrogen). The concentration was measured by Nanodrop One (Thermo Fisher Scientific). The reverse transcription of RNA was conducted using HiScript III 1st Strand cDNA Synthesis Kit (+gDNA wiper) (Vazyme, Nanjing, China). The qPCR was performed using ChamQ SYBR qPCR Master Mix (Vazyme). U6 and GAPDH were used as the internal control respectively for miR-212-5p and lncRNA detection. The relative expression values were calculated using the 2^-ΔΔCt^ method for target genes. The primers for miR-212-5p and U6 were purchased from RiboBio Co., Ltd (Guangzhou, China). The primers for lncRNAs were as follows: DLEU2: sense, GTC TTT TGA AAG GTG TAC TGC AAG, and antisense, TGG TAT CAA AAT TTA CAC TAG AGC C. DNLZ: sense, TCG TCT ACA CCT GCA AGC CAG, and antisense, CTC TTC CCA TTC AGG TCC GAG. SNAPIN: sense, ACG TAC ACG CCG TCA GAA C, and antisense, GGT TTA GCC GTC TCA GTC GT. CDK2AP1: sense, ACA CCA CTC GCC ACC ATT TT, and antisense, CTC CGC GTA TTT GCT TTG GG. LMNTD2-AS1: sense, CCT TTG CCT GTG ATG TGG TG, antisense, AGT GAG GCT TCA GTT TGG GG. SNHG5: sense, GAA GAT GCA AAG ATA CAC G, antisense, CAA CAG TCA AGT AAA CCT CG. FGD5-AS1: sense, CTC GCT TAG CCA AGG CTT CT, antisense, ACA AAA AGC ATA TTC TAC AGA CGC T. CMAS: sense, GGA GTT CGT GAA GTG ACC GA, antisense, GAG ATT AGC CTC ACC TTA ATA CTC T. GAPDH: sense, GGG AGC CAA AAG GGT CAT, and antisense, GAG TCC TTC CAC GAT ACC AA.

### Plasmid construction and cell transfection

LncRNA DLEU2 short interfering RNA (siRNAs), miR-212-5p mimic, miR-212-5p inhibitor and their corresponding controls were synthesized by Ribo Co., Ltd (Guangdong, China). SiRNAs and miR-212-5p inhibitor were transfected at a final concentration of 100nM using ExFect^®^ Transfection reagent (Vazyme). The miR-212-5p mimic was transfected at a final concentration of 50nM. The DLEU2 targeting sequences were as follows: siDLEU2-1: GGT GAG AAC TGA CTA AAC T, siDLEU2-2: GGT AGA AGC TTG AAG GAA A, and siDLEU2-3: TGT CAG CAA AGA ACT GTA A. The sequence of DLEU2 and the full-length of ELF3 CDS region were respectively cloned into pcDNA-3.1 vector (Invitrogen). The efficiency of overexpression of DLEU2 or ELF3 was detected by qRT-PCR or western blot.

### Western blot assay

Western blot analysis was performed as previously described^25^. The primary antibodies were as follows: mouse monoclonal anti-E-cadherin (1:2000, Proteintech, Rosemont, USA), mouse monoclonal anti-Vimentin (1:10000, Proteintech), rabbit polyclonal anti-MMP9 (1:500, Proteintech), rabbit polyclonal anti-VEGF (1:1000, Proteintech), rabbit polyclonal anti-ELF3 (1:500, Abcam, Cambridge, MA), rabbit polyclonal anti-Notch3 (1:500, Proteintech). Rabbit polyclonal anti-GAPDH (1:5000, Proteintech) was used as an endogenous control.

### Dual-luciferase reporter assay

The sequence of DLEU2 and ELF3 3'UTR region, which containing the putative or mutative miR-212-5p binding sites was respectively inserted into the downstream of the reporter gene of the pRL-TK vector (Promega, Madison, WI). The above constructed plasmids were confirmed using DNA sequencing. Luciferase reporter assays were conducted in HEK-293T as described previously^25^. In brief, For DLEU2 luciferase reporter assay, pRL-TK-DLEU2 or pRL-TK-DLEU2 mutant vectors and pGL3 control (Promega) were transfected into HEK-293T along with miR-212-5p mimic or miR-212-5p inhibitor by ExFect^®^ Transfection reagent (Vazyme) according to the protocol. For miR-212-5p target gene ELF3 luciferase reporter assay, pRL-TK-ELF3-3΄UTR or pRL-TK-ELF3-3΄UTR mutant vectors and pGL3 control (Promega) were transfected into HEK-293T along with miR-212-5p mimic or miR-212-5p inhibitor. The luciferase activity was detected 48 h post transfection using the Dual-Lumi luciferase reporter gene assay kit (Beyotime).

### Statistical analysis

All statistical analyses were conducted by SPSS 22.0 software. Statistical analysis was performed with Student's *t*-test (for two-group comparisons). All experiments were performed in triplicate and data are presented as the mean ± S.D. A p-value < 0.05 represented statistically significant. *P < 0.05, ** *P* < 0.01, ***P < 0.001.

## Results

### Huaier suppresses cell viability, migration and invasion in A549 and H1299 cells

**Figure 1:**
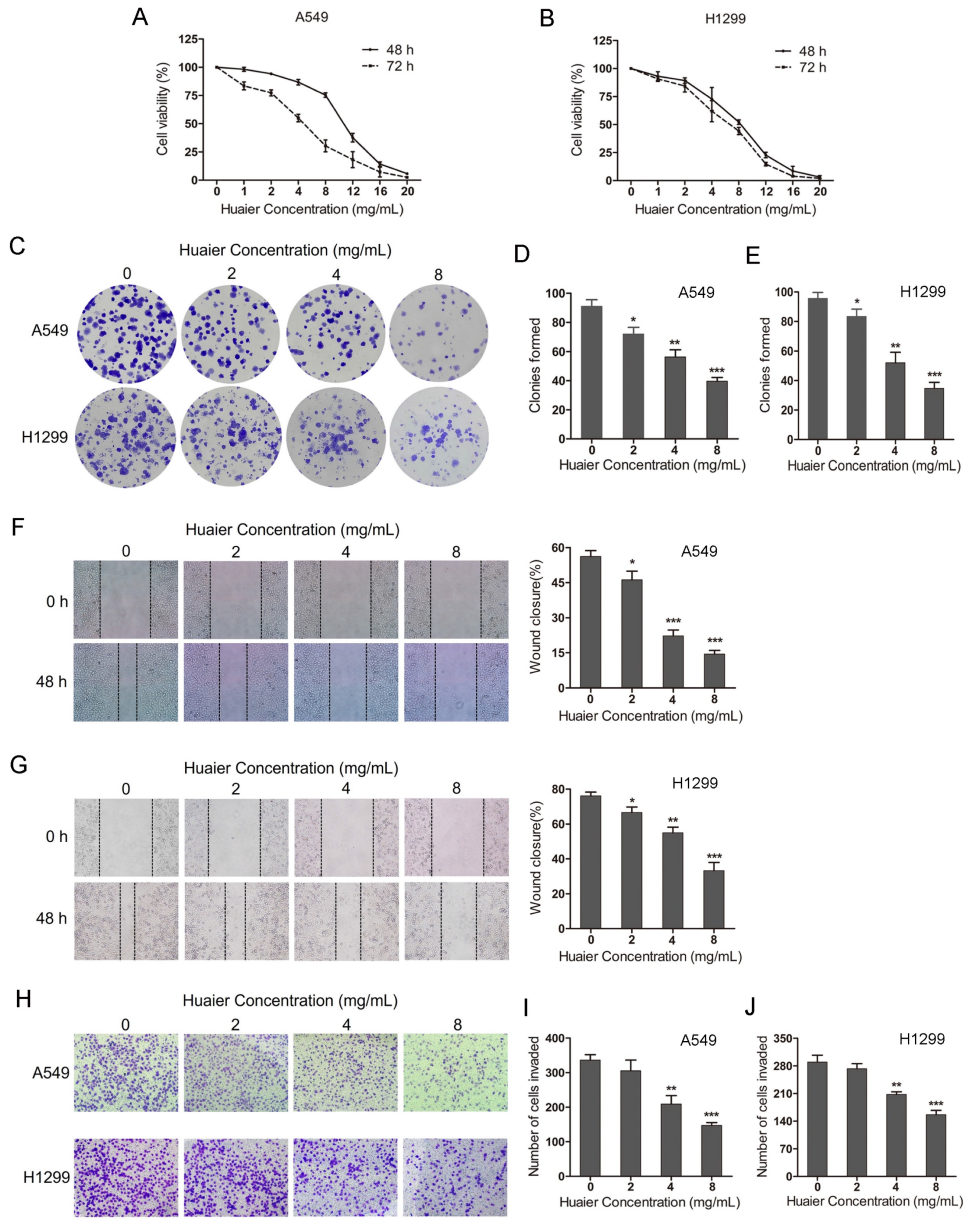
** Effects of Huaier on lung cancer cell viability, migration and invasion.** (A and B) Cell viability of A549 and H1299 cells treated with 0, 1, 2, 4, 8, 12, 16, 20 mg/mL Huaier for 48 h and 72 h. (C, D and E) Colony formation assay for A549 and H1299 cells treated with 0, 2, 4, 8 mg/mL Huaier. (F and G) Wound healing assay reflecting cell migration with 0, 2, 4, 8 mg/mL Huaier treatment for 48 h. (H, I and J) Transwell assay reflecting cell invasion with 0, 2, 4, 8 mg/mL Huaier treatment for 48 h.

To explore the effects of Huaier on NSCLC cells malignant viability, CCK8 assay and colony formation assay were used. The CCK8 assay showed that Huaier treatment (0-20 mg/mL for 48-72 h) inhibited A549 and H1299 cells growth and the inhibitory effects exhibited a concentration- and time-dependent manner (Figure 1A and B). The colony formation assay showed that the colonies were significantly reduced in A549 and H1299 cells with the increased concentration of Huaier treatment (2, 4, 8 mg/mL) compared with the control group (Figure 1C, D and E). Moreover, the wound healing assay suggested that Huaier treatment obviously decreased the migration of NSCLC cells (Figure 1F and G). The transwell assay demonstrated that the invaded cells were markedly decreased after Huaier treatment (4, 8 mg/mL) compared with the control group, while 2 mg/mL Huaier treatment had no significant effects on cell invasion (Figure 1H, I and J). These results reveal that Huaier suppresses cell viability, migration and invasion in NSCLC cells. According to these results, we chose the treatment (4 mg/mL Huaier for 48 h) as the optimal concentration and time for the subsequent experiments.

### Huaier downregulates the highly expressed lncRNA DLEU2 in NSCLC cell lines

**Figure 2:**
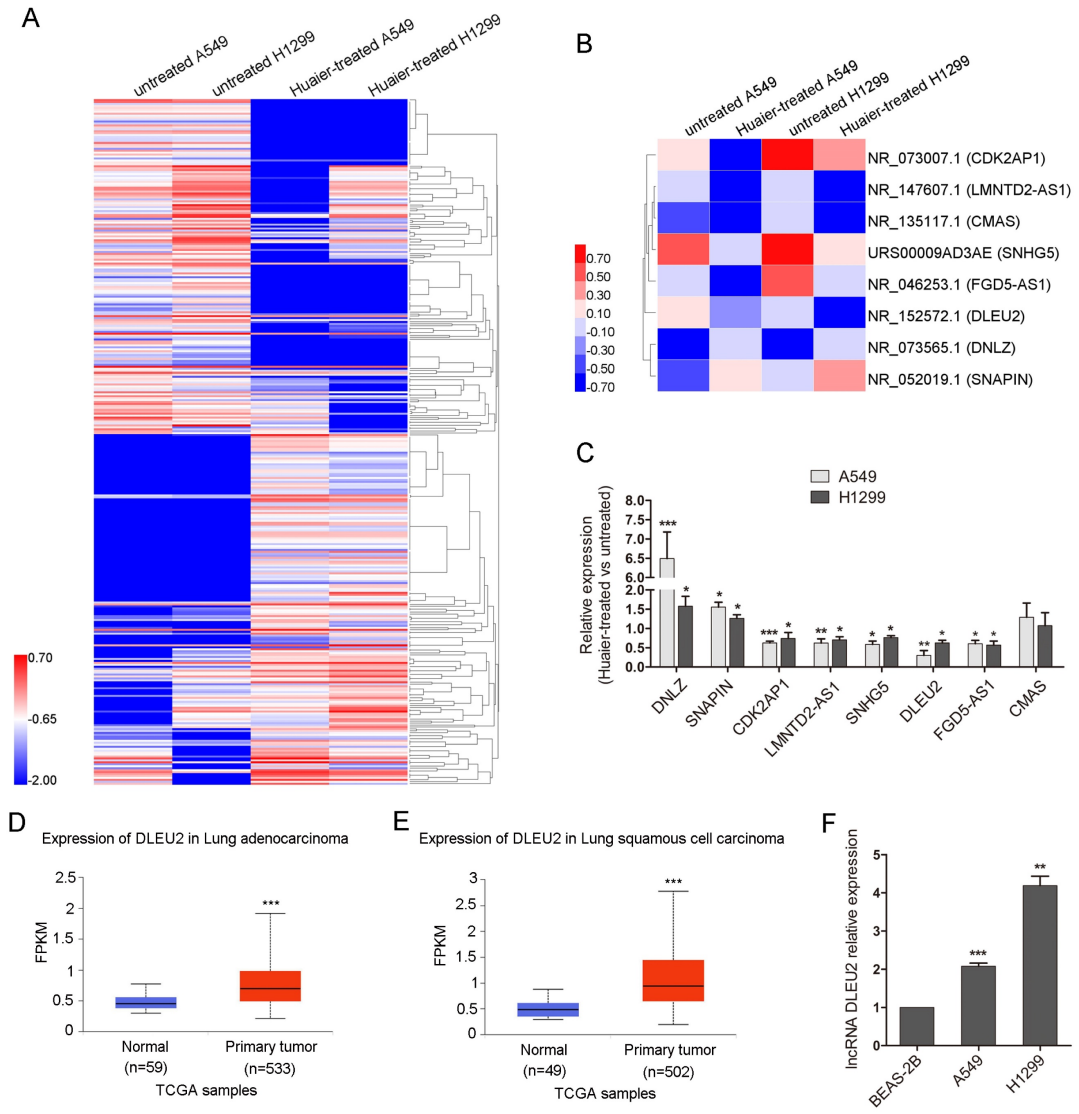
** The overexpressed lncRNA DLEU2 in lung cancer cell lines was downregulated after Huaier treatment.** (A) Heatmap showing the significantly co-upregulated and co-downregulated lncRNAs of A549 and H1299 cells treated with 4 mg/mL Huaier for 48 h. The lncRNA expression profile was based on Huaier-treated versus untreated A549 and H1299 cells at fold change > 2 and false discovery rate (FDR) < 0.001. Red represented high expression and blue represented low expression. (B) Heatmap displaying the indicated differentially expressed lncRNAs. Six co-downregulated lncRNAs (CDK2AP1, LMNTD2-AS1, CMAS, SNHG5, FGD5-AS1, DLEU2) and two co-upregulated lncRNAs (DNLZ, SNAPIN). (C) The qRT-PCR to verify the co-upregulated and co-downregulated lncRNAs. (D and E) DLEU2 expression in lung adenocarcinoma and lung squamous carcinoma compared to the noncancerous lung tissues from UALCAN cancer database. (F) The qRT-PCR for DLEU2 expression in NSCLC cells and the noncancerous lung epithelial cell line BEAS-2B.

To investigate the mechanisms by which Huaier suppresses cell viability, migration and invasion in NSCLC cells, lncRNA sequencing was conducted. Heatmap showed 180 known significantly co-upregulated and 172 co-downregulated lncRNAs of 4 mg/mL Huaier-treated A549 and H1299 cells compared with the untreated group (Figure 2A). Heatmap also partially displayed six co-downregulated lncRNAs (CDK2AP1, LMNTD2-AS1, CMAS, SNHG5, FGD5-AS1, DLEU2) and two co-upregulated lncRNAs (DNLZ, SNAPIN) (Figure 2B). The qRT-PCR was used to verify the significantly differently expressed lncRNAs in Figure 2B. The expression of the selected lncRNAs by qRT-PCR assay was consistent with the results of lncRNA sequencing, while lncRNA CMAS exhibited no difference (Figure 2C). The occasional inconsistency may be related to the different detection principles of qRT-PCR and lncRNA sequencing. Among the differently expressed lncRNAs, Deleted in lymphocytic leukemia 2 (DLEU2) has been reported to be related to the tumorigenesis and progression of various malignant tumors and the upregulation of DLEU2 expression was associated with poor survival in patients with NSCLC^26^, ^27^. So, we chose DLEU2 for the further study. We analyzed the expression of DLEU2 in lung cancer tissues in UALCAN cancer database^28^. In UALCAN cancer database, DLEU2 expression was higher in 533 lung adenocarcinoma tissues than in 59 noncancerous lung tissues (Figure 2D). So did that in 502 lung squamous carcinoma tissues compared to 49 noncancerous lung tissues (Figure 2E). DLEU2 was also highly expressed in A549 and H1299 cells than in BEAS-2B (Figure 2F). These results suggest that Huaier can inhibit the overexpressed DLEU2 in NSCLC cell lines.

### Huaier suppresses NSCLC cells viability, migration and invasion partially by downregulating DLEU2

**Figure 3:**
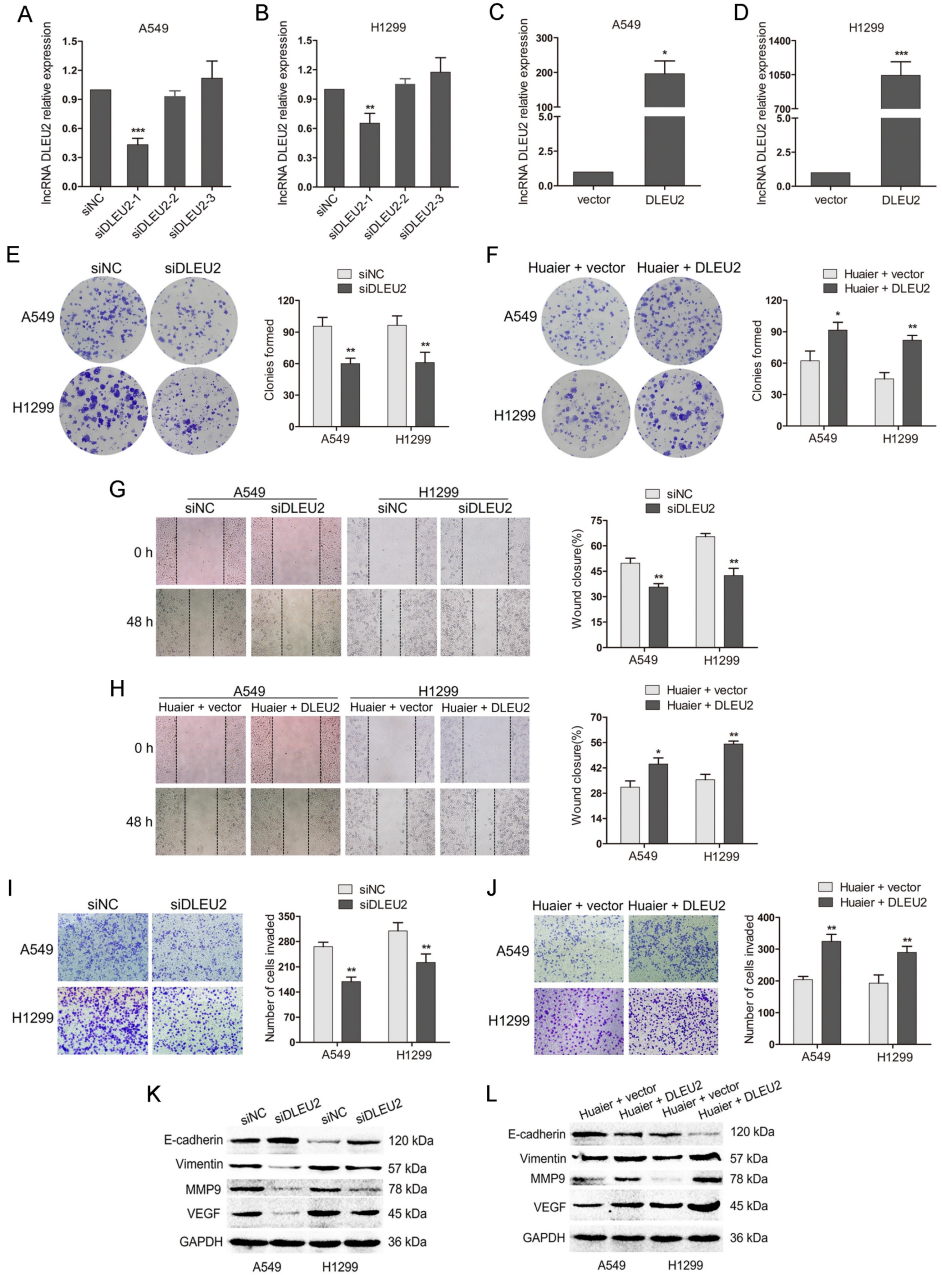
**The effects of inhibition or overexpression of DLEU2 on cell viability, migration and invasion in both A549 and H1299 or Huaier-treated A549 and H1299 cells.** (A and B) The qRT-PCR assay for DLEU2 expression in A549 and H1299 cells transfected with DLEU2 siRNA (siDLEU2-1, siDLEU2-2, siDLEU2-3) or control (siNC). (C and D) The qRT-PCR to detect the DLEU2 level in NSCLC cells transfected with pcDNA3.1-DLEU2 (DLEU2) or vector control (vector). (E, G, I and K) A549 and H1299 cells transfected with DLEU2 siRNA (siDLEU2) or control (siNC) for 48 h. Colony formation assay reflected cell viability (E). Wound healing assay reflected cell migration (G). Transwell assay reflected cell invasion (I). Western blotting detected the protein expression of E-cadherin, Vimentin, MMP9 and VEGF (K). (F, H, J and L) A549 and H1299 cells treated with 4 mg/mL Huaier and pcDNA3.1-DLEU2 (Huaier + DLEU2) or Huaier and vector control (Huaier + vector) for 48 h. Colony formation assay (F), wound healing assay (H), transwell assay (J) and western blot assay(L) were performed.

To further investigate the functions of DLEU2 in NSCLC cells, DLEU2 siRNAs (siDLEU2-1, siDLEU2-2, siDLEU2-3) were transfected into A549 and H1299 cells. The qRT-PCR showed that DLEU2 siRNA (siDLEU2-1) markedly inhibited DLEU2 expression, while siDLEU2-2 and siDLEU2-3 showed no effects (Figure 3A and B). Therefore, siDLEU2-1 was employed in the subsequent experiments to interfere with DLEU2 expression. In addition, DLEU2 was obviously increased in A549 and H1299 cells transfected with pcDNA3.1-DLEU2 compared with control (Figure 3C and D). We then transfected A549 and H1299 cells with siDLEU2 or siNC. DLEU2 silencing resulted in decreased colony formation, slower wound healing and less invaded cells in NSCLC cells (Figure 3E, G and I). DLEU2 silencing also led to the upregulation of the epithelial marker E-cadherin and the downregulation of mesenchymal marker Vimentin, the cell matrix metalloproteinase MMP9 and the vascular endothelial growth factor VEGF (Figure 3K). We further transfected pcDNA3.1-DLEU2 or control into Huaier-treated A549 and H1299 cells. The reduced colony formation medicated by Huaier treatment was reversed by DLEU2 overexpression (Figure 3F). Wound healing and transwell assays demonstrated that Huaier treatment-mediated inhibition in migration and invasion of both A549 and H1299 cells were overturned by DLEU2 overexpression simultaneously (Figure 3F, H and J). The western blot assay showed that the upregulation of E-cadherin and the downregulation of Vimentin, MMP9, VEGF by Huaier treatment were inverted in A549 and H1299 cells by highly expressed DLEU2 (Figure 3K). Collectively, these results suggest that inhibition of DLEU2 restrain NSCLC progression and Huaier suppresses NSCLC cells viability, migration and invasion partially by downregulating DLEU2.

### MiR-212-5p is the downstream of DLEU2 and miR-212-5p inhibition weakens the anti-tumor effects of DLEU2 downregulation and Huaier treatment

**Figure 4:**
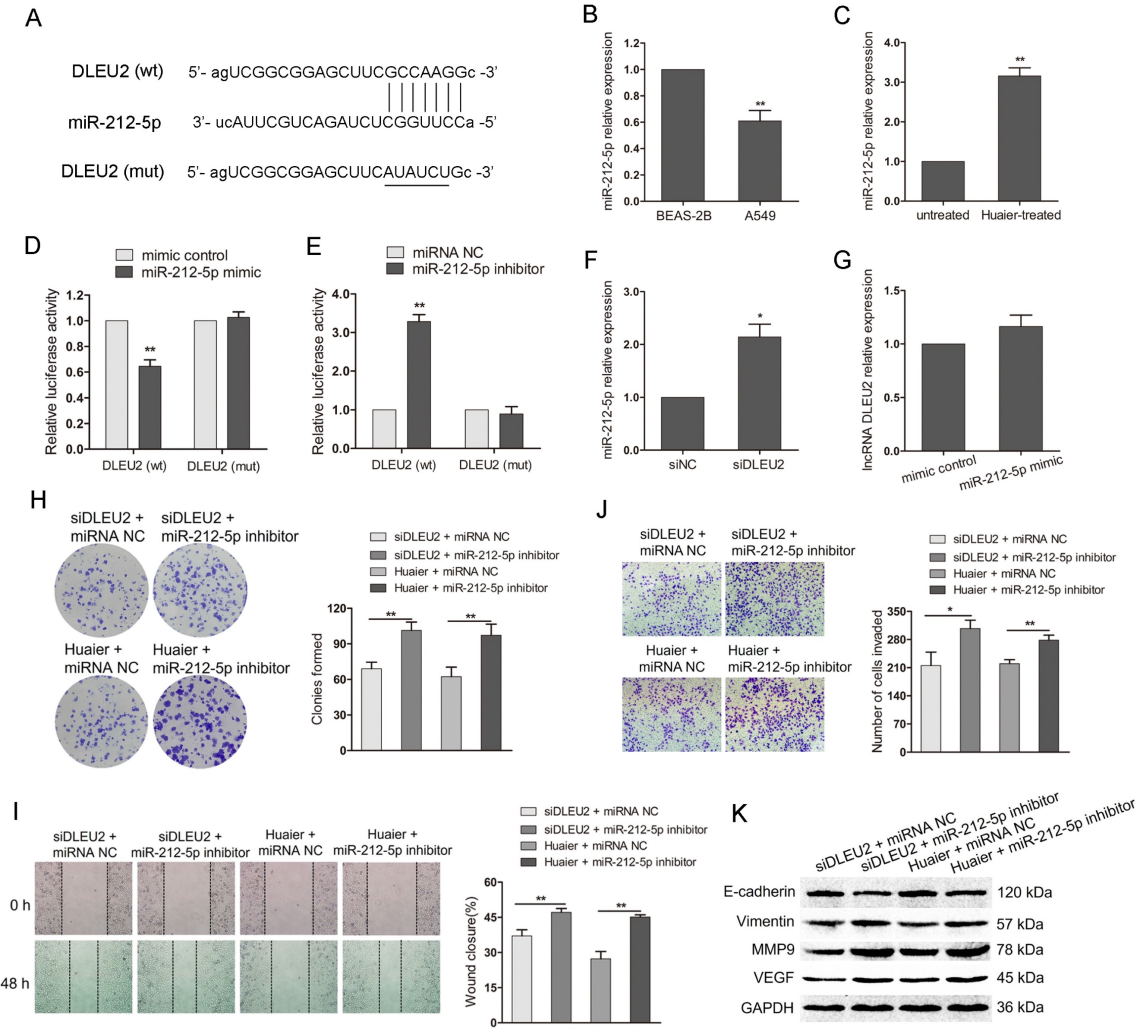
**The interaction between DLEU2 and miR-212-5p in the anti-tumor effects of Huaier.** (A) DLEU2 wild-type (wt) sequence containing the putative miR-212-5p binding sites and DLEU2 sequence with the mutant (mut) miR-212-5p binding sites. (B) The miR-212-5p level in A549 cells and the noncancerous lung epithelial cell line BEAS-2B. (C) The miR-212-5p level in Huaier-treated and untreated A549 cells. (D and E) Luciferase reporter assay in HEK-293T. HEK-293T was cotransfected with pRL-TK carrying wild-type or mutant DLEU2 sequence and the miR-212-5p mimic or miR-212-5p inhibitor, and the luciferase activity was detected 48 h after transfection. (F) The miR-212-5p level in NSCLC cells transfected with DLEU2 siRNA (siDLEU2) or control (siNC). (G) The expression of DLEU2 in NSCLC cells transfected with miR-212-5p mimic or mimic control. (H) Colony formation assay in DLEU2-silenced A549 cells transfected with miR-212-5p inhibitor (siDLEU2 + miR-212-5p inhibitor) or inhibitor control (siDLEU2 + miRNA NC). Colony formation assay in Huaier-treated A549 cells transfected with miR-212-5p inhibitor (Huaier + miR-212-5p inhibitor) or inhibitor control (Huaier + miRNA NC). (I, J and K) A549 cells were treated as in (H). wound healing assay (I), transwell assay (J) and western blot assay (K) were performed.

DLEU2 has been reported to involve in the tumorigenesis by playing as competing endogenous RNAs (ceRNAs) through competitively binding miRNAs^27^, ^29^. We then predicted miRNA that possibly bound to DLEU2 using starBase v3.0 (http://starbase.sysu.edu.cn/). The research showed that miR-212-5p could bind to DLEU2 with high conservation (Figure 4A). The qRT-PCR indicated that miR-212-5p was low expressed in A549 cells compared with BEAS-2B (Figure 4B).

Additionally, miR-212-5p was upregulated in Huaier-treated A549 cells (Figure 4C). The level of miR-212-5p and DLEU2 exhibited the opposite expression in NSCLC cells or Huaier-treated NSCLC cells. To further clarify whether miR-212-5p could directly bind to DLEU2, we constructed luciferase reporters containing DLEU2 sequence with either wild-type or mutant miR-212-5p binding site. The luciferase reporter assay in HEK-293T showed that the overexpression of miR-212-5p negatively regulated the luciferase activity in a DLEU2 wild-type construct but not in a mutant (Figure 4D). Inhibition of miR-212-5p obviously increased the luciferase activity with wild-type DLEU2 sequence but not the mutant (Figure 4E). Furthermore, the expression of miR-212-5p was significantly increased with DLEU2 inhibition in A549 cells (Figure 4F). But, the expression of DLEU2 was not obviously changed by miR-212-5p overexpression in A549 cells (Figure 4G). These results suggest that there is a direct binding between miR-212-5p and DLEU2 and miR-212-5p is the downstream of DLEU2.

To explore the involvement of miR-212-5p in the anti-tumor effects of Huaier mediated by DLEU2, we cotransfected DLEU2 siRNA and miR-212-5p inhibitor or DLEU2 siRNA and inhibitor control into A549 cells. At the same time, we also transfected miR-212-5p inhibitor or control into Huaier-treated A549 cells. As shown in Figure 4H, the decreased colony formation caused by DLEU2 inhibition or Huaier treatment was reversed after cotransfection with miR-212-5p inhibitor. Wound healing and transwell assay showed that, in the presence of miR-212-5p inhibitor, the wound closure was increased and the invaded cells were much more despite DLEU2 inhibition or Huaier treatment (Figure 4I and J). The downregulation of E-cadherin and the upregulation of Vimentin, MMP9 and VEGF were induced in group (siDLEU2 + miR-212-5p inhibitor, Huaier + miR-212-5p inhibitor) compared to the respective control group (Figure 4K). These findings suggest that the inhibition of miR-212-5p attenuates the anti-tumor effects of DLEU2 downregulation and Huaier treatment, highlighting the pivotal role of DLEU2/miR-212-5p in mediating the anti-tumor effects of Huaier.

### DLEU2, miR-212-5p and ELF3 constitutes a ceRNA network and DLEU2/miR-212-5p/ELF3 axis mediates the anti-tumor effects of Huaier

**Figure 5:**
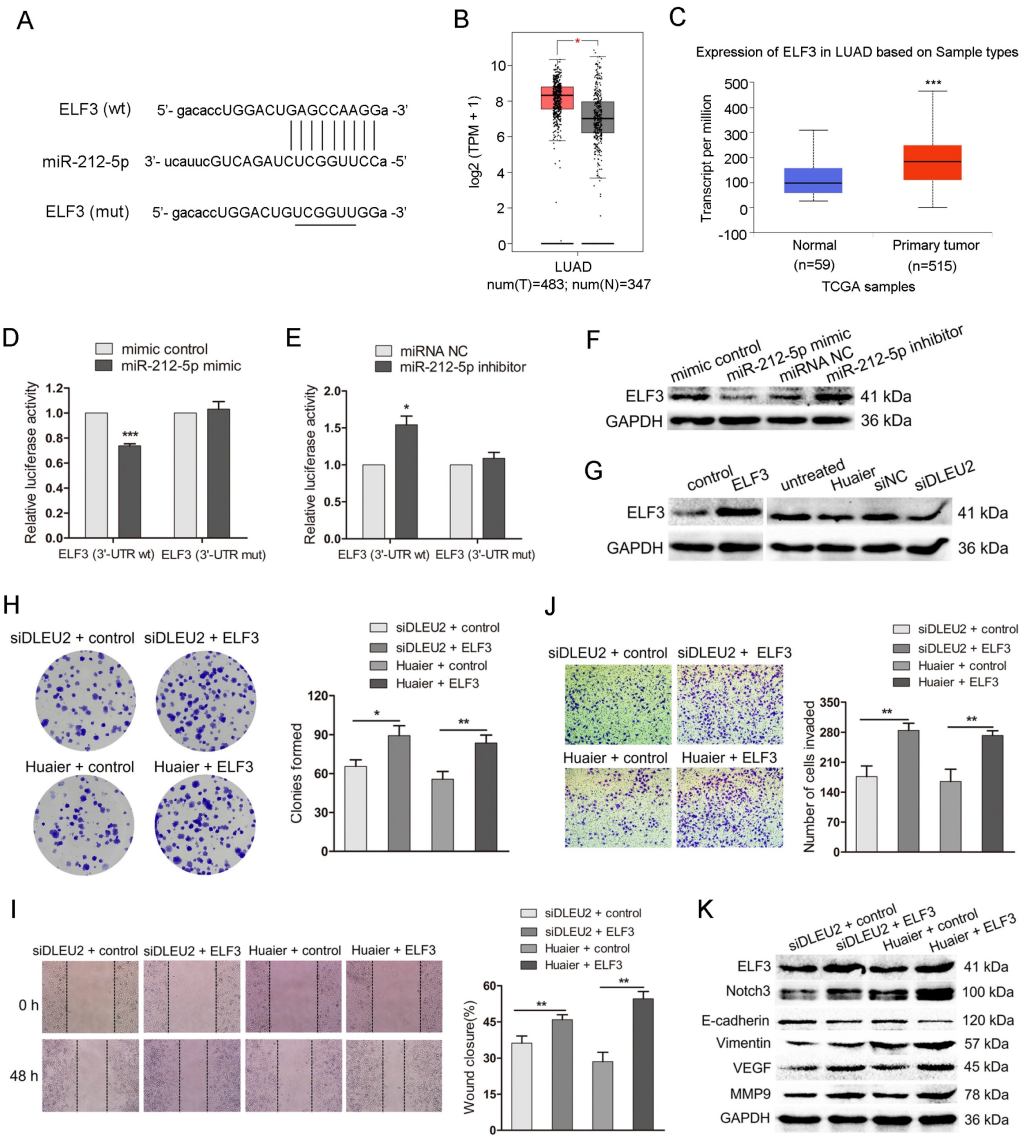
** The DLEU2/miR-212-5p/ELF3 axis in the anti-tumor effects of Huaier.** (A) ELF3 3'UTR wild-type (wt) sequence containing the putative miR-212-5p binding sites and ELF3 3'UTR sequence with the mutant (mut) miR-212-5p binding sites. (B) ELF3 expression in lung adenocarcinoma compared to the noncancerous lung tissues from GEPIA database. (C) ELF3 expression in lung adenocarcinoma from UALCAN cancer database. (D and E) Luciferase reporter assay in HEK-293T. HEK-293T was cotransfected with pRL-TK carrying wild-type or mutant ELF3 3'UTR sequence and the miR-212-5p mimic or miR-212-5p inhibitor, and the luciferase activity was detected 48 h after transfection. (F) The western blot assay for ELF3 in A549 transfected with miR-212-5p mimic or miR-212-5p inhibitor. (G) The western blot assay for ELF3 in A549 cells transfected with pcDNA3.1-ELF3 (ELF3), DLEU2 siRNA (siDLEU2) or Huaier and the respective control. (H) Colony formation assay in DLEU2-silenced A549 cells transfected with pcDNA3.1-ELF3 (siDLEU2 + ELF3) or vector control (siDLEU2 + control). Colony formation assay in Huaier-treated A549 transfected with pcDNA3.1-ELF3 (Huaier + ELF3) or vector control (Huaier + control). (I and J) A549 cells were treated as in (H). wound healing assay (I), transwell assay (J) were performed. (K) The western blot assay for ELF3, Notch3, E-cadherin, Vimentin, MMP9 and VEGF in A549 cells treated as in (H).

To further elucidate the underlying biological mechanisms mediated by DLEU2/miR-212-5p in Huaier-treated NSCLC cells, we searched for the potential targets of miR-212-5p by using TargetScan 8.0 (https://www.targetscan.org) and miRDB (http://www.mirdb.org/) database. MiR-212-5p was predicted to have putative binding to E74 Like ETS Transcription Factor 3 (ELF3) 3′UTR in both TargetScan8.0 and miRDB (Figure 5A). ELF3 acts as an oncogene and a promising target for therapeutic intervention in lung adenocarcinoma, with genetic, functional, and clinical evidence that supports the specificity of its subtypes^30^. ELF3 also promotes cell growth and metastasis in non-small cell lung cancer via the PI3K/Akt and ERK signaling pathways^31^, ^32^. In UALCAN cancer database, the expression of ELF3 was significantly elevated in lung adenocarcinoma tissues (n = 483), compared to the noncancerous lung tissues (n = 347; Figure 5B). Besides, GEPIA database also indicated that ELF3 level was higher in lung adenocarcinoma tissues (n = 515) than in normal control tissues (n = 59) (Figure 5C). The luciferase reporter assay in HEK-293T exhibited that miR-212-5p mimic repressed the luciferase activity from ELF3 wild-type 3′ UTR, but not from mutant 3′UTR (Figure 5D). Conversely, when miR-212-5p was downregulated, there was a significant elevation in luciferase activity in HEK-293T with the wild-type 3′UTR, but not the mutant (Figure 5E). These results indicated that ELF3 was a direct target of miR-212-5p. In A549 cells, miR-212-5p mimic significantly inhibited ELF3 protein level, while miR-212-5p inhibitor led to the increased protein level (Figure 5F). Moreover, the transfection with pcDNA3.1-ELF3 obviously upregulated the ELF3 protein level, while Huaier treatment or siDLEU2 transfection resulted in the decreased the protein level in A549(Figure 5G). These results suggested that ELF3 expression was regulated by the miR-212-5p, DLEU2 and Huaier and DLEU2, MiR-212-5p and ELF3 constitute a ceRNA network.

To clarify the ELF3 in the anti-tumor effects of Huaier mediated by DLEU2/MiR-212-5p, we cotransfected DLEU2 siRNA and pcDNA3.1-ELF3 or DLEU2 siRNA and vector control into A549 cells. Meanwhile, we also transfected pcDNA3.1-ELF3 and vector control into Huaier-treated A549 cells. The colony formation assay showed that the colonies were increased in group (siDLEU2 + ELF3, Huaier + ELF3) compared with that in the control group (Figure 5H). As shown in Figure 5I and J, the impaired wound closure and reduced cell invasion induced by DLEU2 siRNA or Huaier treatment were restored upon co-transfection with pcDNA3.1-ELF3. DLEU2-silenced and Huaier-treated A549 cells transfected with pcDNA3.1-ELF3 exhibited elevated protein levels of ELF3, Notch3, Vimentin, MMP9 and VEGF, as well as reduced E-cadherin level (Figure 5K). The findings suggest that the DLEU2/miR-212-5p/ELF3 axis plays a crucial role in mediating the anti-tumor effects of Huaier.

## Discussion

Lung cancer is the leading cause of cancer-related mortality, with metastasis being a significant contributor to treatment failure in clinical patients^33^. The exploration of more optimized drugs and the development of more suitable strategies are still necessary to enhance the survival rate and expand treatment options for lung cancer. TCM, characterized by unique pharmacological mechanisms, minimal side effects and low toxicity, has demonstrated efficacy against lung cancer^34^. We have previously reported that Huaier inhibits proliferation and induces apoptosis in human lung cancer cells via a miR-26b-5p-EZH2-mediated approach^25^. LncRNAs play biological role in NSCLC through different pathways, such as lncRNA-protein interaction, lncRNA-ceRNA network, and binding to promoter regions of encoding genes^35^. Since lncRNAs are implicated in the pathogenesis of NSCLC, it remains to be determined whether they contribute to the anti-NSCLC effects of Huaier. This is the inaugural article to document that lncRNA serves as a mediator of Huaier's anti-tumor effects in NSCLC.

In the present study, we discovered Huaier inhibited the viability, migratory capacity, and invasive potential of NSCLC cells in a concentration- and time-dependent manner. LncRNA DLEU2 was significantly downregulated in NSCLC cells after Huaier treatment. DLEU2 was initially identified through a comprehensive analysis of the chromosome 13q14 region, a genomic locus that is frequently subjected to deletions in B-cell chronic lymphocytic leukemia (BCLL)^36^. Subsequent investigations have demonstrated the upregulation and oncogenic effects of DLEU2 across various cancer types^37^. DLEU2 captured our attention, prompting us to focus on this particular lncRNA. Moreover, DLEU2 exhibited high expression levels in primary lung tumors and cancer cells. Inhibition of DLEU2 in A549 and H1299 cells resulted in a significant decrease in cell viability, migration, and invasion. Conversely, overexpression of DLEU2 in Huaier-treated NSCLC cells counteracted the anti-tumor effects of Huaier, leading to increased colony formation capacity, migratory potential, and invasive ability.

Emerging evidence suggests that DLEU2 may disrupt miRNA function and their interplay is involved in the regulation of numerous types of cancer. We then identified miRNAs that bound to DLEU2. We discovered that StarBase V3.0 predicted an interaction between miR-212-5p and DLEU2. MiR-212-5p functions as a tumor-suppressor in various cancers, such as prostate cancer^38^, breast cancer^39^, and so on. However, miR-212-5p is also reported to be a tumor-promoter in certain cancers, such as colorectal cancer^40^ and lung adenocarcinoma cells^41^. The expression and function of miR-212-5p in cancers exhibit inconsistency across different genetic contexts and specific cell type. In our study, miR-212-5p was low expressed in A549 cells and was then upregulated after huaier treatment. Luciferase reporter assay showed that miR-212-5p directly bound to DLEU2. MiR-212-5p functions as a downstream effector of DLEU2, and inhibition of miR-212-5p attenuates the anti-tumor effects elicited by downregulation of DLEU2 and treatment with Huaier. The DLEU2/miR-212-5p pathway mediates the anti-tumor effects of Huaier.

To investigate the mechanisms mediated by DLEU2/miR-212-5p in Huaier-treated NSCLC cells, we identified a miR-212-5p target gene, ELF3. ELF3 was found to have a direct interaction with miR-212-5p and its expression was regulated by both miR-212-5p and Huaier, as well as DLEU2. These findings elucidated a complex ceRNA network involving DLEU2, miR-212-5p, and ELF3. Moreover, the upregulation of ELF3 counteracted the inhibitory effects induced by DLEU2 knockdown or Huaier treatment, leading to enhanced colony formation, migration and invasion in NSCLC. The protein kinase C iota activates the level of Notch3 via phosphorylating the transcription factor ELF3 and subsequent occupancy of ELF3 on the Notch3 promoter in lung adenocarcinoma^42^. The Notch3 upregulation facilitates the autologous invasion and migration of lung adenocarcinoma cells^43^. The modulation of Notch3 signaling is a pivotal factor in the progression and pathogenesis of lung cancer^44^, ^45^. In the current study, overexpression of ELF3 reversed the effects of DLEU2 silencing and Huaier treatment by promoting Notch3 expression, which subsequently upregulated the downstream proteins Vimentin, MMP9 and VEGF while downregulating E-cadherin. Collectively, the DLEU2/miR-212-5p/ELF3 pathway mediates the anti-tumor effects of Huaier by ultimately regulating proteins involved in migration and invasion.

**Figure 6:**
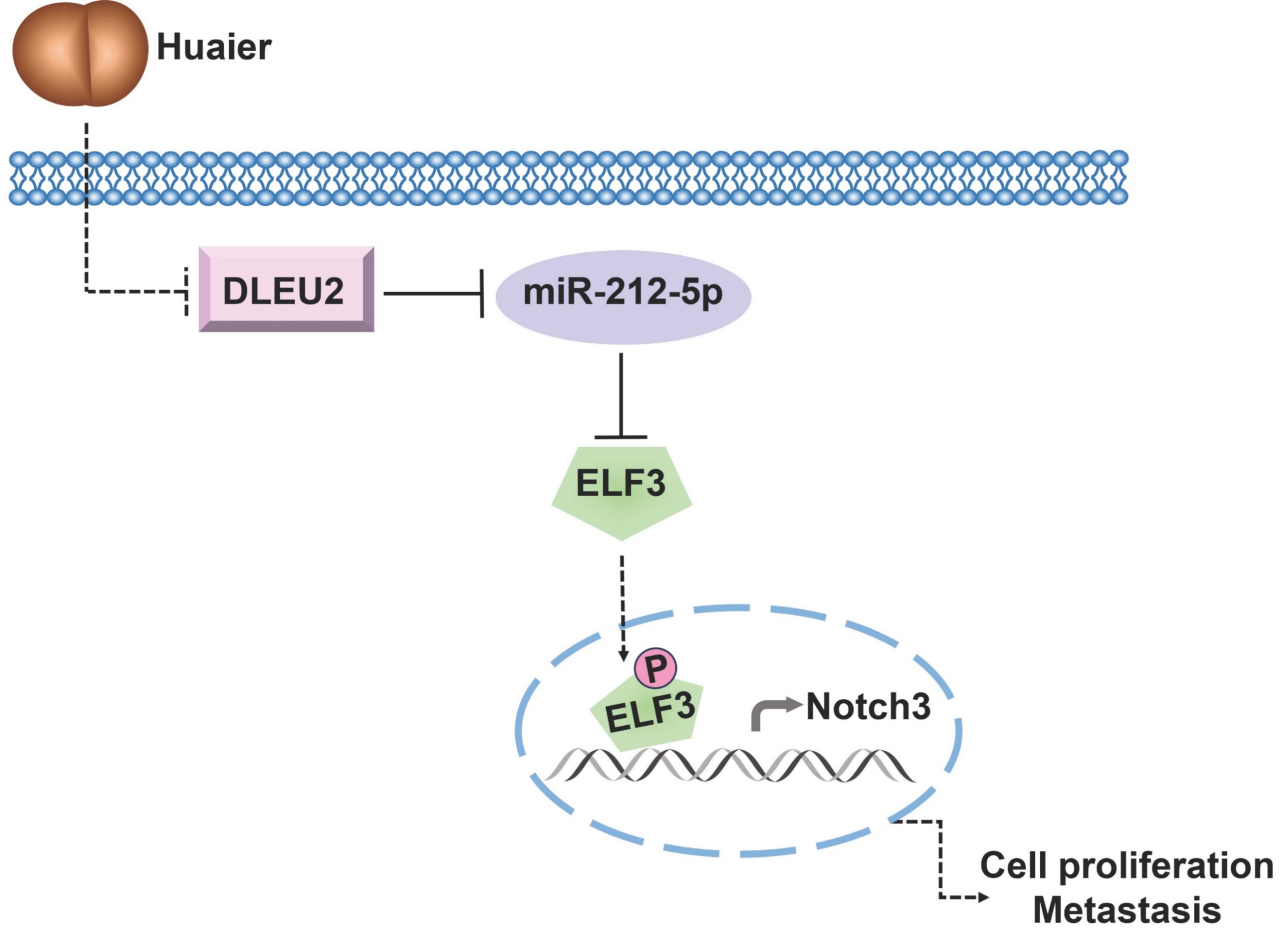
Schematic representation of the underlying mechanisms governing Huaier's anti-tumor effects via modulation of the DLEU2/miR-212-5p/ELF3 signaling pathway.

In conclusion, our study provides novel insights into the underlying mechanism of Huaier's anti-tumor effects in NSCLC. The ceRNA network involving DLEU2/miR-212-5p/ELF3 axis emerges as a crucial signaling pathway through which Huaier effectively suppresses NSCLC viability, migration, and invasion (Figure 6). These findings lay the foundation for considering the DLEU2/miR-212-5p/ELF3 axis as a promising therapeutic target for NSCLC treatment.

## Abbreviations

lncRNAs: long noncoding RNAs
NSCLC: non-small cell lung cancer
DLEU2: Deleted in lymphocytic leukemia 2
ceRNA: competing endogenous RNAs
ELF3: E74 Like ETS Transcription Factor 3
SCLC: small cell lung cancer
TCM: Traditional Chinese Medicine
LINC01140: Long intergenic non-protein coding RNA 1140
HNF1A-AS1: Hepatocyte nuclear factor 1 homeobox A antisense RNA 1
SNHG6: small nucleolar RNA host gene 6
ZNRD1-AS1: zinc ribbon domain-containing 1-antisense 1
HITT: HIF-1α inhibitor at translation level
CSNK2B: Casein kinase 2 beta
ATCC: American Type Culture Collection
HEK-293T: Human embryonic kidney 293T
FBS: fetal bovine serum
CCK8: Cell counting kit-8
PBS: phosphate-buffered saline
qRT‑PCR: quantitative reverse transcription polymerase chain reaction
DNLZ: DNL-type zinc finger
SNAPIN: synaptosomal-associated protein associated protein
CDK2AP1: cyclin dependent kinase 2 associated protein 1
LMNTD2-AS1: lamin tail domain containing 2 antisense RNA 1
SNHG5: small nucleolar RNA host gene 5
FGD5-AS1: PH domain containing 5 antisense RNA 1
CMAS: cytidine monophosphate N-acetylneuraminic acid synthetase
MMP9: matrix metallopeptidase 9
VEGF: vascular endothelial cell growth factor
Notch3: neurogenic locus notch homolog protein 3
PI3K: phosphatidylinositol 3-kinase
Akt: serine/threonine kinase
ERK: extracellular-regulated kinase
EZH2: enhancer of zeste 2 polycomb repressive complex 2 subunit
BCLL: B-cell chronic lymphocytic leukemia
